# The Role of Thymoma and Thymic Hyperplasia as Prognostic Risk Factors for Secondary Generalisation in Adults with Ocular Myasthenia Gravis: A Systematic Narrative Review

**DOI:** 10.22599/bioj.315

**Published:** 2023-11-30

**Authors:** Laura Wilson, Helen Davis

**Affiliations:** 1 NHS Greater Glasgow and Clyde, UK; 2 The medical school University of Sheffield, UK

**Keywords:** Ocular Myasthenia Gravis, Thymoma, Thymic Hyperplasia, Generalisation, Generalised Myasthenia Gravis

## Abstract

**Purpose:**

The conversion of ocular myasthenia gravis (OMG) to generalised myasthenia gravis (GMG) is reported to differ depending on the presence of generalisation risk factors (Mazzoli et al. 2018). Thymic pathology has been recognised as a potential risk factor for generalisation in the literature (Teo et al. 2017). Thymoma and thymic hyperplasia have yet to be examined as a risk factor for generalisation of OMG independently of other risk factors in the literature. Thus, the purpose of this review is to examine the literature to identify whether thymoma and thymic hyperplasia do increase the risk of OMG progressing to GMG.

**Methods:**

A literature search was carried out which employed a systematic approach. The search was undertaken using the following academic libraries: MEDLINE, Embase and Starplus. The search was limited to publications between the years 2001 to 2021. The search yielded 82 studies, which after the screening of titles and abstracts, left 62 studies for further analysis against the inclusion and exclusion criteria.

**Results:**

The review found thymoma to be associated with an increased risk of GMG development. However, there was a scarce amount of literature which investigated thymic hyperplasia. Therefore, a firm conclusion could not be made with regards to thymic hyperplasia and the risk of GMG development.

**Conclusions:**

This review provides evidence for the consideration of thymectomy early after thymomatous OMG diagnosis to prevent GMG conversion. As the review did not collect enough evidence to support the influence of thymic hyperplasia on OMG conversion, further research is required.

## Introduction

Generalised myasthenia gravis (GMG) is a rare organ specific autoimmune condition characterised by post synaptic disturbance at the neuromuscular junction situated at the motor end plate (Mygland et al. 2000). The disease is initiated by antibodies, most commonly binding to the nicotinic acetylcholine receptor (AChRs) (Karni et al. 2016). Other antibodies have been identified such as muscle specific kinase (MuSK), LRP-4 Titin, Agrin and clustered antibodies to the AChR and to MuSK (Hoch et al. 2001; Higuchi et al. 2011). Primarily myasthenia gravis (MG) causes weaknesses of the bulbar, proximal, respiratory, and ocular muscles with typical features of inherent variability and fatigability (Gilhus et al. 2016).

Ocular myasthenia gravis (OMG) is a subtype of MG, and is classified as muscle weakness which is restricted to the extraocular, levator palpebrae superioris, and orbicularis oculi muscles (Nair et al. 2014). Ocular myasthenia gravis manifests clinically as intermittent and variable diplopia, ptosis, and orbicularis weakness (Luchanok & Kaminski 2008).

### Role of the thymus gland

Thymus involvement in OMG should be suspected in cases with AChR positive antibodies as the thymus holds all the required components to initiate AChR antibody responses (Cavalcante et al. 2011). The thymus is documented to have pathological changes in the majority of AChR positive cases and a significant number of patients who receive thymectomy demonstrate clinical improvement (Gilhus et al. 2019).

### Thymoma and thymic hyperplasia

A thymoma is a rare and typically benign tumour that is derived from thymic epithelial cells and bordered by T cells (Yamada et al. 2020). Thymoma’s have been suggested to be derived from cortical epithelial cells and do not have the functional medulla where antigen presenting cells participate in negative selection (Okumura et al. 2008). Therefore, it is proposed that thymoma produce autoreactive T cells, initiating autoimmunity reducing tolerance to AChR and thus, driving thymoma mediated AChR positive MG (Gilhus et al. 2019).

Thymic hyperplasia is the term used to describe hyperplastic follicles within the thymus (Zaĭrat’iants 1991). The primary feature of thymic hyperplasia is the growth of ectopic germinal centre-sites of B cell maturation and development, which are non-existent in the normal thymus (Priola & Priola 2014). The reduction in tolerance to the AChR along with the presence of B cells results in the production of AChR-antibodies (Berrih-Aknin & Le Panse 2014).

Thymectomy is indicated in patients with thymoma, and is effective in the treatment of GMG with thymic hyperplasia (Gilhus & Verschuuren 2015; Gilhus et al. 2019). The role of thymectomy in the treatment of OMG continues to be debated (Li et al. 2020). Li et al. (2021) proposed that thymectomy (in cases of thymoma and thymic hyperplasia) when carried out early in pure OMG; postpones, reduces the risk, or even prevents secondary progression to GMG, but controversy remains (Hendricks et al. 2019). Li et al. (2021) revealed statistical significance in a cohort of 519 (with 131 participants undergoing thymectomy) where thymectomy independently reduced the rate of conversion to GMG; 95 participants had thymoma and 28 had thymic hyperplasia (adjusted HR: 0.41, 95% CI 0.25–0.66, P < 0.001). The findings from Liu et al. (2011) are in line with Li et al. (2021) with their study of 110 participants with OMG who underwent thymectomy for thymoma, revealing that none of their cohort progressed to GMG post thymectomy. Similarly, Sommer et al. (1997) also found that thymectomy resulted in good outcomes in OMG, however, unlike the other authors they did not find any benefit of thymectomy over medical management alone.

### Conversion from OMG to GMG

The rate of conversion after initial diagnosis has been reported to vary between 30–80% within the first two years (Apinyawasisuk et al. 2020). This conversion rate is described as a result of the presence or absence of prognostic risk factors, such as female sex, positive anti-AChR antibodies, and later onset of disease (Kamarajah et al. 2018).

Thymoma and thymic hyperplasia have been investigated and identified as predictive factors for generalisation in OMG, however there is some controversy in the literature. Teo et al. (2017) and Nagia et al. (2015) are both in agreement that the presence of thymoma indicated an increased risk for the development of GMG. Furthermore, Wang et al. (2017) published significant results for a relationship between thymic hyperplasia and conversion to GMG within the first six months after diagnosis. Hong et al. (2008) also disclosed a positive correlation between generalisation and thymoma but found no relationship in participants with thymic hyperplasia. However, Hendricks et al. (2019) demonstrated insignificant results for the influence of thymoma on conversion to GMG.

The main purpose of this review is to identify, isolate and evaluate the evidence published surrounding the prognostic impact of thymoma and thymic hyperplasia in patients with OMG. The knowledge gained may assist in the understanding of the clinical course of OMG in patients with thymoma and thymic hyperplasia and thus, aid future management plans.

## Methods

### Data sources and extraction

The review was limited to studies written in the English language and published between the years 2001 and 2021. A twenty-year timescale was chosen to allow for a sufficient data search but rule out significantly outdated treatment practices. The literature search was completed using MEDLINE, Embase, and Starplus. The key words used in the search to identify the studies are listed in Table 1. All studies included were observational cohort studies.

**Table 1:** Key words used as search terms to identify each component of the research question.


POPULATION	EXPOSURE	OUTCOME

Ocular Myasthenia Gravis	Thymoma	Generalisation

Myasthenia Gravis	Thymic Hyperplasia	Conversion

Positive Acetylcholine Antibodies	Thymic Pathology	Progression

Thymic Abnormalities	Development	


### Inclusion criteria

All participants had to be aged ≥18 years with a diagnosis of OMG as defined by the diagnostic criteria proposed by Osserman (1967). The presence of abnormal thymic pathology must have been confirmed by thymus imaging by either magnetic resonance imaging (MRI) scan or computerised tomography (CT) scan, with or without supportive histopathology. The participants had to meet Osserman (1967) diagnostic criteria to confirm progression from OMG to generalised myasthenia gravis.

### Exclusion criteria

Review articles, editorials, letters to editors and case reports were all excluded. Studies that were not reported in English or peer reviewed were also excluded. Participants that had a primary diagnosis of GMG who were not sub-grouped appropriately were excluded. Studies that had not stated if investigation for thymic pathology were made by either CT/MRI scan or by pathology report following thymectomy were excluded.

### Quality assessment

Analysis of the relevance and quality of the studies was performed using STROBE (Strengthening the Reporting of Observational Studies in Epidemiology) reporting checklist for all studies including observational studies. The Scottish Intercollegiate Guidelines Network (SIGN) cohort study checklist was implemented for critical appraisal of cohort studies (Health Improvement Scotland 2021). While grading scales can provide an overall grading score for each study, these were not included. This was due to the vast variability between the weighting given to methodology between scales and because many scales fail to consider the direction of bias.

Out of the 33 points set by the STROBE checklist, 31 were applied to the studies included in this review, and out of the 16 points set by the SIGN setlist, 11 were applicable. The scores of each were converted into percentages. As per the guidance set by SIGN, as all of the above are retrospective cohort studies (RCS) and are therefore a weaker design, they cannot receive a rating higher than an ‘acceptable’. All nine studies were deemed ‘acceptable’ by SIGN standards in minimising the risk of bias and were therefore accepted into the systematic narrative review.

## Results

**Figure 1:**
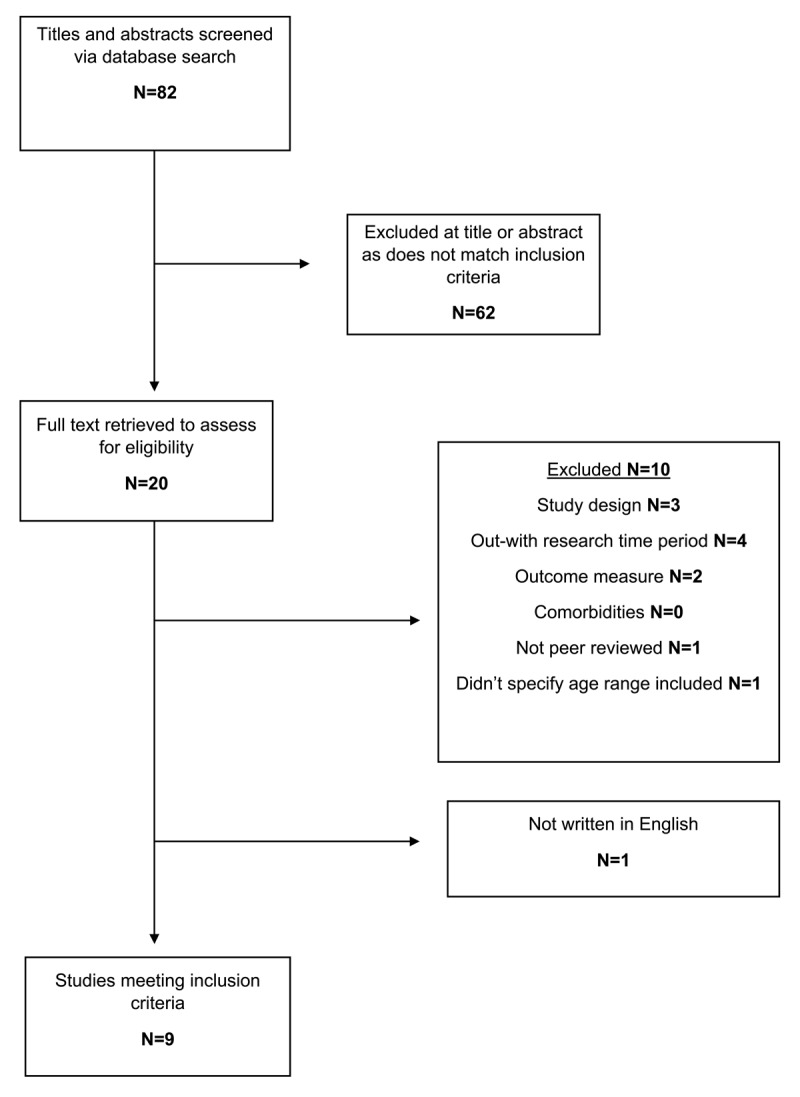
Flow diagram demonstrating data selection process using a modified diagram as recommended by PRISMA (Moher et al. 2009).

The Embase, MEDLINE, and Starplus search generated 82 studies. Titles and abstracts of these studies were screened to decide if they met the inclusion criteria. This resulted in 62 studies being excluded. The remaining full 20 studies were requested and read to assess for suitability (Figure 1). Nine studies met all of the inclusion criteria for systematic review (Tables 2 and 3).

**Table 2:** Demographics of RCS Investigating Thymoma and Thymic Hyperplasia.


STUDY	SAMPLE SIZE WITH OMG (N=)	AGE MEDIAN (RANGE)	NO. WITH THYMOMA	NO. WITH THYMIC HYPERPLASIA	FOLLOW-UP MEDIAN MONTHS (RANGE)	COUNTRY

Nagia et al. (2015)	158	61.5(18–85)	8 (5.1%)	0	60.5 (24–300)	USA

Kemchoknatee et al. (2021)	155	49.3 (NS)	30 (19.4%)	0	>4 (NS)	Thailand

Li et al. (2018)	180	NS	35 (19.4%)	56 (31.1%)	23.6 (NS)	Germany

Hong *et al*. (2008)	202	>20 (NS)	33 (18.3%)	18 (10%)	11.8 (NS)	Korea

Kisabay et al. (2022)	139	54.4 (20–97)	9 (10.5%)	6 (7.1%)	56.2 (24–696)	Turkey

Wang et al. (2017)	40	39.8 (4–74)	10 (25%)	22(55%)	Up to 24(NS)	China

Hendricks et al. (2019)	33	59 (NS)	3 (9%)	0	91 (17–333)	USA

Teo et al. (2017)	155	59 (NS)	12 (7.74%)	2 (1.3%)	40.8 (NS)	Singapore


Abbreviations: NS: Not specified. TH: Thymic Hyperplasia. LR: Logistic regression. * Significant when correlation with corticosteroid use.

**Table 3:** Demographics of RCS investigating ‘unspecified’ thymic abnormalities.


STUDY	SAMPLE SIZE	AGE MEDIAN (RANGE)	NO. WITH THYMIC PATHOLOGY	FOLLOW-UP MEDIAN MONTHS (RANGE)	COUNTRY

Apinyawasisuk et al. (2020)	71	52.4 years(NS)	19 (27%)	4.91 years (NS)	Thailand


Three studies used CT or MRI scans to diagnosis thymic pathology in all their participants (Nagia et al. 2015; Kisabay et al. 2022; Kemchoknatee et al. 2021). In a study by, 89% of participants had a CT scan of the thymus. Two studies (Wang et al. 2017; Teo et al. 2017) included participants who all had abnormal thymic pathology diagnosed by CT or MRI scan, with a proportion of their cohort also having a post-thymectomy histology report completed. Two studies did not detail whether CT or MRI was used to diagnose thymic pathology in their cohort (Apinyawasisuk et al. 2020; Hendricks et al. 2019).

### Generalisation in thymoma

Six studies (67%) presented statistically significant results for thymoma increasing the risk of generalisation. Hendricks et al. (2019) – (HR: 2.48, 95% CI 0.7–8.71, P = 0.160) and Wang et al. (2017) demonstrated insignificant results. The statistical analysis for the latter study was not detailed. One study presented borderline findings with no difference seen on regression analysis (HR:1.28; 95% CI 0.58–2.78 p = 0.542) (Kemchoknatee et al. 2021). See Tables 4 and 5 for further details.

**Table 4:** Results of RCS Investigating Thymoma and Thymic Hyperplasia.


STUDY	STATISTICS USED	SIGNIFICANT FOR CONVERSION TO GMG	STATISTICAL RESULTS	QUALITY ANALYSIS SCORE

Nagia et al. (2015)	Cox proportional Hazard model for Univariate and multivariate LR Kaplan-Meyer Estimation	Thymoma: Yes	3.90 [95% CI, 0.86–17.59] p = 0.09	STROBE: 25/31 (80%) SIGN: 9/11

Kemchoknatee et al. (2021)	As Above	Thymoma: No	1.28 [95% CI 0.58–2.78] p = 0.54	STROBE: 24/31 (77%) SIGN:10 /11

Li et al. (2018)	As Above	Thymoma: Yes TH: Yes*	1.66 [95% CI 1.52–2.62] p = 0.029 0.41 [95% CI 0.18–0.95] p = 0.038	STROBE: 28/31 (90%) SIGN: 10/11

Hong et al. (2008)	As Above	Thymoma: Yes TH: No	2.32 [95% CI 1.21–4.45] p = 0.01 NS	STROBE: 27/31 (87%) SIGN: 9/11

Kisabay et al. (2022)	As Above	Thymoma: Yes TH: Yes	3.48 [95% CI 1.8–6.71] p = 0.001 2.98 [95% CI 1.38–5.12] p = 0.005	STROBE: 25/31 (80%) SIGN:10/11

Wang et al. (2017)	Correlation Matrix Multivariate LR	Thymoma: No TH: Yes	NS 71.4% vs. 16.7%, p = 0.027	STROBE: 23/31 (74%) SIGN: 10/11

Hendricks et al. (2019)	Cox proportional hazards Kaplan-Meyer	Thymoma: No	2.48 [95% CI 0.7–8.71] P = 0.16	STROBE: 25/31 (80%) SIGN:10/11

Teo et al. (2017)	Cox proportional Hazard model for Univariate and multivariate LR Kaplan-Meyer Estimation	Thymoma: Yes	HR: 6.88 P = 5 0.009	STROBE: 28/31 (90%) SIGN: 10/11


Abbreviations: NS: Not specified. TH: Thymic Hyperplasia. LR: Logistic regression. * Significant when correlation with corticosteroid use.

**Table 5:** Results of RCS investigating ‘unspecified thymic pathology’.


STUDY	STATISTICS USED	SIGNIFICANT FOR CONVERSION TO GMG	STATISTICAL RESULTS	QUALITY ANALYSIS SCORE

Apinyawasisuk et al. (2020)	Cox proportional Hazard model for Univariate and multivariate LR Kaplan-Meyer Estimation	Unspecified Thymic Pathology: Yes	1.82 [95% CI 0.91–3.67]	STROBE: 28/31 (90%) SIGN: /11


### Cohort size

Differences in cohort sizes were observed within the studies (Table 2). Hendricks et al. (2019) included 33 participants with OMG, 15 (45%) remaining ocular, and 18 (55%) developing GMG. However, only three (9%) of these participants were diagnosed with thymoma. Similarly, Wang et al. (2017) had a small cohort size of 40 participants, with only 10 detected to have thymoma. In comparison, Hong et al. (2008) included 33 (18.3%) participants with thymoma and revealed significant results with thymoma being detected more commonly in the group which converted to GMG than the pure ocular group (36.2% vs. 12.0%, p < 0.001, χ2 test). Also, Li et al. (2018) incorporated 35 (19.4%) participants with thymoma and revealed statistically significant results to suggest thymoma as a risk factor for GMG development (HR: 1.659, 95% CI (1.52–2.617), P = 0.029). As both Hong et al. (2008) and Li et al. (2018) studied sizeable samples with thymoma and both demonstrated that thymoma increases the risk of GMG development, the reliability of the findings in Hendricks et al. (2019) and Wang et al. (2017) are questionable. It can be argued that it is difficult to draw sufficient conclusion surrounding GMG in thymoma with cohort sizes with thymoma as little as three and 20 participants. Furthermore, alike to Hong et al. (2008) and Li et al. (2018), including more participants with thymoma could have impacted the final outcomes of these studies.

### Thymus imaging

The extent of radiological thymic investigation within the cohorts may also have contributed to some of the differing findings. All of the participants within Kisabay et al. (2022) study had chest imaging by either CT or MRI scan. Likewise, Teo et al. (2017) described a positive relationship between thymoma, and GMG occurrence (HR: 6.88 P = 0.009) and 136/155 of the participants included had CT thorax imaging- with thymoma diagnosed in 21 (15.4%). Further, 15 participants had thymectomy and a post-op histological report. Li et al. (2021) who demonstrated a significant relationship in thymoma and conversion, performed a post-operative thymic histological report in the entirety of the cohort. Thus, there was no dubiety surrounding the accuracy of thymic pathology diagnosis of all the participants included in this study. In contrast, Hendricks et al. (2019) who reported statistically insignificant results for a relationship between thymoma and GMG occurrence, omitted to inform the reader what proportion of the cohort had thymus investigation. Failing to include this information, creates some doubt regarding the true number of participants with thymoma in this sample and whether the imaging test used was adequate enough to differentially diagnose thymic pathology sufficiently.

### Generalisation in thymic hyperplasia

Four (44%) of the studies included examined the impact of thymic hyperplasia. Three studies demonstrated significant results for thymic hyperplasia increasing the possibility of generalisation. As well as thymoma, Kisabay et al. (2022) also demonstrated significant findings for thymic hyperplasia posing a risk for conversion to GMG – (HR: 2.980; 95% CI 1.380–5.120 p = 0.005). Wang et al. (2017) has similar findings to Kisabay et al. (2022), suggesting that thymic hyperplasia could play a substantial role in the progression to GMG. Li et al. (2018) found thymic hyperplasia in 56 (31.1%) of their cohort and established a significant outcome that thymic hyperplasia was associated with an increased risk of generalisation in their cohort of participants who had received corticosteroid treatment (HR: 0.414, 95% CI (0.180–0.951), P = 0.038). Nonetheless, Hong et al. (2008) demonstrated contradictory results to the above studies, observing thymic hyperplasia more frequently in their cohort that remained ocular than the cohort who generalised to GMG (11.3% vs. 6.4%). Thus, unveiling no correlation between thymic hyperplasia and conversion rate to GMG.

### Follow-up time

Generalisation can arise at any stage of the disease, therefore, adequate follow-up time of participants under observation is required for an accurate measurement of conversion rate. Follow-up time amid the studies detailed above is inconsistent. Kisabay et al. (2022) and Wang et al. (2017) who suggested thymic hyperplasia is a risk factor for GMG development, both had sufficient follow-up times to allow for generalisation development (Table 2). Kisabay et al. (2022) set a minimum follow-up time of two years as part of the inclusion criteria with a mean of 56.2 months and a range of (24–696 months) and Wang et al. (2017) followed participants for six months to 24 months post OMG diagnosis. Contrary to these two studies, Hong et al. (2008) had a much shorter follow-up time with a mean of 11.8 months with no range specified by the authors. Restricting the follow-up time to less than one year is likely to have created an underestimation of the number of participants who went on to develop GMG. A longer follow-up period, for example, a minimum of two years, may have allowed more time for GMG to develop in participants with thymic hyperplasia converting to GMG and consequently, a different outcome may have been observed from the study.

### Treatment

There is variability in the treatment options given to the participants between the studies and the details provided to the reader concerning dosages and duration of treatments. Wang et al. (2017) and Hendricks et al. (2019) were the only two studies which did not provide detailed information regarding the percentage of participants receiving which type of treatment. Cholinesterase Inhibitors were the most popular treatment of choice for six studies (Kemchoknatee et al. 2021; Li et al. 2018; Hong et al. 2008; Kisabay et al. 2022; Teo et al. 2017; Apinyawasisuk et al. 2020). This was followed by steroid treatment and azathioprine and mycophenolate mofetil (Nagia et al. 2015; Wang et al. 2017; Hendricks et al. 2019). Thymectomy was also employed in six studies (Nagia et al. 2015; Kemchoknatee et al. 2021; Li et al. 2018; Hong et al. 2008; Wang et al. 2017; Teo et al. 2017).

#### Thymectomy

Six studies included participants who had thymectomy at some stage in their treatment (Nagia et al. 2015; Hong et al. 2008; Wang et al. 2017; Teo et al. 2017; Li et al. 2018; Kemchoknatee et al. 2021). Nagia et al. (2015) performed thymectomy in all eight participants with thymoma. Twenty-seven participants with thymoma had thymectomy in the Hong et al. (2008) study. Sixty-one participants with thymoma or thymic hyperplasia had thymectomy in the Wang et al. (2017) study. Teo et al. (2017) carried out thymectomy in 15 participants with suspected thymoma and Kemchoknatee et al. (2021) employed thymectomy in 15 participants with thymoma. Despite thymectomy being a strong confounding factor when assessing the effect of thymoma and thymic hyperplasia on generalisation, none of the above studies accounted for thymectomy in their statistical analysis. Thus, there is a risk-modifying effect of thymectomy on generalisation. Moreover, with the exception of Li et al. (2018), the above studies did not provide details regarding the timing of thymectomy, more specifically, they did not stipulate if the participants had thymoma before or after the development of GMG. As documented by studies such as Li et al. (2021) thymectomy has been demonstrated as a useful treatment option in the reduction or prevention of generalisation, hence, the timing of thymectomy is crucial in the disease process. Therefore, failing to inform the reader of the timing of the thymectomy within the patient’s journey makes it difficult to extrapolate if the thymectomy influenced the progression to GMG. It can be deliberated that the difference in the relationship between thymic pathology and the conversion rate demonstrated between the studies could be a result of thymectomy being carried out at varying time scales. For example, immediately after thymoma or thymic hyperplasia is diagnosed or carried out one-year post diagnosis. Carrying out thymectomy immediately post diagnosis could prevent the impact of thymic pathology in GMG development and postponing thymectomy could allow time for thymic pathology to trigger conversion.

Li et al. (2018) was the only study to perform thymectomy on the entire cohort and the only study to specify a timescale for thymectomy in respect to generalisation. All 180 participants in the Li et al. (2018) study had thymectomy and within the cohort of 110 participants developed GMG. Nineteen of these participants had a thymectomy prior to generalisation with a median delay of eight months (range 1–24 months) after symptom onset, whilst 91 had thymectomy after conversion with a median delay of 18 months (range 1–120) after symptom onset. By specifying the timescale of thymectomy, the authors have accentuated the impact of thymoma and thymic hyperplasia as risk factors for generalisation in their cohort. Delaying thymectomy in participants with thymic hyperplasia and thymoma may have allowed for the conversion to GMG and removing the thymic pathology quickly could have prevented generalisation from taking place.

### Medical management

Early prednisolone and immunosuppressant treatment have been demonstrated to considerably reduce the risk for generalisation when compared to pyridostigmine treatment use alone, specifically in those recently diagnosed with OMG (Li et al. 2019). Furthermore, early immunosuppressant treatment could lessen the impact of thymic pathology in triggering GMG development (Ding et al. 2020). The medical treatment given to the participants within all nine studies may have influenced generalisation rates. However, Nagia et al. (2015) was the only study to detail what fraction of participants with thymoma had immunosuppressant treatment and what percentage had pyridostigmine treatment only. The remaining eight studies failed to enlighten the reader regarding the medical treatment plan of the participants with thymic pathology. Consequently, within these studies, it is unclear whether immunosuppressant treatment was given to participants with thymic pathology and if so, at what stage of the disease process this was given. Thus, the reader cannot consider the influence of treatment on generalisation in the presence of thymic pathology. Li et al. (2018) emphasised the impact of treatment on the conversion rate as this study revealed that participants with thymic hyperplasia who had been treated with corticosteroids had a 58.6% lesser risk of converting to GMG than those who were not treated with corticosteroids. This could be interpreted that the corticosteroids that were prescribed may have weakened the weight of thymic pathology on generalisation rate. There was no statistical relationship between corticosteroid treatment and generalisation rate in participants with thymoma or participants with a normal thymus.

There are also differences in the amount of treatment given between the studies which may have influenced the generalisation rates found in those with thymoma. Some studies included participants who were treated with cholinesterase inhibitors and steroids while other studies were comprised of participants who were given combination immune therapy (steroidal and non-steroidal immunosuppressants) and cholinesterase inhibitors. It has also been demonstrated that combination therapy is associated with a decrease in the risk of conversion to GMG when compared to single immunotherapy treatment (Ruan et al. 2022). However, this theory does not fit with the studies included in this review, as three studies, (Teo et al. 2017; Li et al. 2018; Hong et al. 2008) all had participants who were prescribed combination immunosuppressant treatment, and all determined positive correlations in thymoma and conversation to GMG. While Wang et al. (2017) employed single immunosuppressant treatment and Apinyawasisuk et al. (2020) prescribed pyridostigmine only. It could be considered that combination treatment does not impact generalisation in participants with thymic pathology compared to those with a normal thymus however further research would be required to validate this. With the exception of thymectomy, specific treatment in OMG with thymic pathology in the prevention of GMG is vastly undocumented.

## Discussion

This is the first systematic narrative review to independently investigate the role of thymoma and thymic hyperplasia as prognostic risk factors in OMG generalisation. The review disclosed a positive relationship between thymoma and OMG conversion to GMG. This conclusion was made based on the fact that the majority of studies had positive findings for generalisation in OMG with thymoma and those who did not lacked validity. This was due to insufficiencies in the reporting of methodological approaches and small sample sizes. However, the treatment modalities were heterogenous (both medical and thymectomy) among the studies, therefore, making it difficult to derive sound conclusions.

This review demonstrates a rarity in the amount of literature which examined the influence of thymic hyperplasia on generalisation. While the scarce literature that was uncovered does show a trend for a positive relationship between thymic hyperplasia and conversion to GMG, with such limited evidence, a confident conclusion for the role of thymic hyperplasia in generalisation could not be produced. As a result of strict age inclusion criteria, Wong et al. (2016) randomised controlled study designed to create prognostic risk score for generalisation was excluded from the review. The study did however explore thymic hyperplasia as a predictor for generalisation. The authors concluded with a univariate logistic regression that thymic hyperplasia in AChR positive participants was weakly correlated with GMG development. Thus, while the review was restricted to four studies examining thymic hyperplasia, other studies exist to support its role in GMG risk.

The outcome of this review is significant when considering that some patients with thymoma can present with only ocular symptoms and early thymectomy may avoid the mortality and morbidity of GMG. Furthermore, the review could be important when considering the value of thymectomy in preventing generalisation. This is a small narrative review, comprising only nine studies. The lack of studies suitable for inclusion is likely a result of a lack of data, not only due to the rarity of the OMG, but an insufficiency of clinical information due to the delay in referral to neurology before generalisation occurs. As stated previously, all studies investigated several risk factors for conversion to GMG within one study. Therefore, despite attempts to control confounding variables with multivariate analysis, bias is inevitable. This is particularly prominent with the medical treatment implemented, as discussed in the critical analysis of the studies.

The British guidelines for myasthenia gravis advise thymectomy in OMG in those with positive AChR antibodies younger than 45 years as this can increase the probability of remission, reduce the risk of generalisation, and reduce the need for corticosteroids (Sussman et al. 2015). This systematic narrative review is useful as it is of great importance that risk factors for GMG development are known and that those at clinical risk of conversion to GMG are identified quickly and thymectomy performed. Thus, this systematic narrative review could contribute to crucial decision making in the management of OMG as it supports the British guidelines for the benefit of removing thymoma in reducing the likelihood of generalisation.

Despite British guidelines, thymectomy in purely OMG is not yet widely executed in treatment (Li et al. 2021). This could partly be a product of the fact the improvement in symptoms and quality of life is outweighed by the prospect and risks of undertaking a significant surgery such as thymectomy. Also, it may be a result of neuro-ophthalmologists being apprehensive to recommend a major surgery to improve symptoms like diplopia or clinical signs like ptosis. While less invasive surgical techniques for thymectomy are optional, patients must be counselled that it is not guaranteed that minimally invasive approaches will have the same results as extended transsternal thymectomy (Gronseth et al. 2020).

Several studies have investigated and recommended that thymectomy improved remission rates in OMG in non-thymomatous participants (Zhu et al. 2017; Mineo & Ambrogi, 2013; Nakamura et al. 1996). Evidence to evaluate the use of thymectomy in the treatment of OMG in those with thymoma is limited, however, it is particularly scarce in thymic hyperplasia. Past studies which have measured the benefit of thymectomy in thymic pathology have exhibited that it is useful in generating remission of the disease. Roberts et al. (2001) evaluated the efficiency of thymectomy in the treatment of OMG in 61 participants, with 12 of these presenting with thymoma. Thirty-three percent of the cohort with thymoma were deemed to be cured and 33% showed clinical improvement, however, this did not reach significance (p < 0.39). The authors hinted that their data could suggest that thymectomy could prevent generalisation, however, they do not go on to specify with what findings they came to that conclusion. An older study by Evoli et al. (1988) suggested against thymectomy in non-thymomatous participants with OMG, but in their cohort of 84 participants with thymoma, they uncovered a marginally better clinical course if thymectomy was performed quickly after OMG.

The value of implementing thymectomy after OMG to slow or stop GMG development has been explored somewhat in the literature. As introduced earlier in this review, Li et al. (2021) produced the first retrospective cohort study which investigated the effectiveness of thymectomy in inhibiting generalisation in OMG in participants with a mean age of 45.4 years old. Despite documenting that participants with thymoma (n = 95) and thymic hyperplasia (n = 28) underwent thymectomy in their sample, the authors did not include thymic hyperplasia as a variable in their logistic model. While the study provides robust evidence for thymectomy in preventing GMG in thymomatous OMG participants, it does not explore or justify thymectomy in thymic hyperplasia. Similarly, Liu et al. (2011) detected thymoma in five participants and thymic hyperplasia in 106 participants who underwent thymectomy but only included thymoma in their analysis. While the study demonstrated no significance on the outcome of OMG in thymoma, none of the cohort progressed to GMG within the median follow-up time of 33.5 months. The authors suggested therefore that thymectomy may be more successful in halting disease progression but did highlight that a larger prospective study would be necessary to compare these treatment options. However, it must be stressed that this study included a wide age range of participants from 7–73 years. As this study incorporated paediatrics, its results may not be fully pertinent to the results of this review.

Li et al. (2018) not only emphasised that thymoma was a risk factor in the conversion of OMG to GMG, but also that performing thymectomy early after diagnosis could aid in deterrence of GMG.As discussed earlier in the review, thymectomy was performed on the full cohort and in those with thymoma, generalisation was observed more when thymectomy was delayed by a mean of 18 months when compared to those when it was delayed only by a mean of eight months after diagnosis. Thus, this study provides good rationale for thymectomy as a treatment option in thymoma or thymic hyperplasia. Moreover, this study consisted of participants with both early and late onset OMG, which is a contrast to Li et al. (2021), which median age is considered early onset OMG (<50 years of age).

This systematic narrative review was not able to identify an adequate amount of evidence to make a concrete assumption of the role of thymic hyperplasia in OMG disease progression and likewise, there is a significant lack of literature which suggests a role of thymectomy in the prevention of OMG in thymic hyperplasia. Nevertheless, as this review’s results did demonstrate an inclination that thymic hyperplasia can play a part as a risk factor for generalisation, this may open up an opportunity for further investigation in this area as it highlights some intriguing questions. Firstly, this review leaves question regarding the role of thymic hyperplasia in OMG progression. Secondly it raises the question if thymectomy is also beneficial in reducing the risk of generalisation if the thymic hyperplasia is removed in the same way demonstrated in thymoma. As thymectomy is shown to be of use in generalisation prevention in thymoma and non-thymomatous cases, it is possible that this may be no different in the case of thymic hyperplasia. However, further research would be necessary to explore this.

### Limitations of the review

All studies included were of a retrospective cohort design, recognised as a lower quality study design. Pure systematic reviews are characteristically composed of solely randomised controlled trials; thus, this review was more representative of a narrative review. Inevitably, each study was carried out independently from each other by different examiners, methods, and populations. The impact of this bias is highlighted in the inconsistency in methods of thymic investigation, as discussed in the review of the literature. Furthermore, with all studies being of retrospective cohort design there was no standardisation in evaluation criteria. Therefore, it must be assumed by the reader that all participants included in the studies have been diagnosed correctly with either solely OMG or GMG. Also, as this was a retrospective chart review of clinical records, studies had to exclude some participants due to missing records, further reducing their sample size.

Another limitation is that the review did not exclude studies with a less than two-year follow-up time. The influence of this is also shown to create heterogeneity in results amongst studies, as demonstrated in the review of the literature. Moreover, this review did not identify any UK studies. Lastly, due to the date parameters, the review was also subject to bias as it excluded any studies published before 2001 and after 2021. Thus, the review omits to contain all of the literature published and the most up to date research.

## Conclusion

In conclusion, the review found thymoma to be a possible risk factor for secondary generalisation of OMG. This was conclusion generated by the six studies that demonstrated positive results and had good methodology. However, three of the included studies did not help reaching this conclusion due to methodological flaws including small cohort sizes, poorly reported methodology, and inadequate description of the type of thymic imaging performed. The review only identified four studies that investigated thymic hyperplasia, with three of those indicating that thymic hyperplasia is a risk factor for generalisation. This review was unable to make assumptions surrounding the influence of thymic hyperplasia and secondary generalisation. Furthermore, methodological weaknesses were also detected in these studies, such as insufficient follow-up time.

Further research is encouraged as no randomised controlled studies were incorporated. Due to the conclusions derived, a multicentre investigation that includes a large cohort of patients with thymoma and a standardised outline of diagnostic criteria, substantial follow-up time and sufficient thymus imaging is required. Moreover, as this review did not gather sufficient evidence to determine the weight of thymic hyperplasia on the clinical progression of OMG, further research is required in this area.

Lastly, further research is required to ascertain how beneficial early thymectomy is in preventing GMG when the risk factor of thymoma is present. A prospective, randomised controlled trial would be the most desirable study design in order to clearly establish the value of thymectomy. Moreover, future study should aim to clarify the most favourable timescale for carrying out thymectomy to ensure patients remain purely OMG. Further studies could authenticate the recommendations made by the change model of this review and so, shape the management of newly diagnosed OMG.
